# Fundamental properties and principal areas of focus in antibody–drug conjugates formulation development

**DOI:** 10.1093/abt/tbaf005

**Published:** 2025-03-09

**Authors:** Lili Wen, Yuanyuan Zhang, Chenxi Sun, Shawn Shouye Wang, Yuhui Gong, Chunyuan Jia, Jianjun Luo

**Affiliations:** Department of Bioconjugate Product Development and Manufacturing, WuXi XDC Co., Ltd., Wuxi, Jiangsu, P.R. China; Department of Bioconjugate Product Development and Manufacturing, WuXi XDC Co., Ltd., Wuxi, Jiangsu, P.R. China; Department of Bioconjugate Product Development and Manufacturing, WuXi XDC Co., Ltd., Wuxi, Jiangsu, P.R. China; Business Enablement North America, XDC ConjuTech USA LLC, Middletown, DE, United States; Department of Bioconjugate Product Development and Manufacturing, WuXi XDC Co., Ltd., Wuxi, Jiangsu, P.R. China; Department of Bioconjugate Product Development and Manufacturing, WuXi XDC Co., Ltd., Wuxi, Jiangsu, P.R. China; Department of Bioconjugate Product Development and Manufacturing, WuXi XDC Co., Ltd., Wuxi, Jiangsu, P.R. China

**Keywords:** antibody–drug conjugate, formulation, stability, drug product development, drug product manufacturing

## Abstract

Antibody–drug conjugates (ADCs) have emerged as a rapidly expanding class of therapeutics driven by their superior specificity and clinical efficacy. 14 out of 16 commercially approved ADCs are formulated as lyophilized forms because ADC is generally considered to be less stable than unmodified antibody. The formulation development for ADCs, particularly liquid formulation, presents unique challenges due to their intricate structural complexity, physicochemical properties, and degradation pathways. This review provides the first comprehensive analysis of formulation strategies employed in commercial ADCs. Furthermore, this review discusses the key areas of focus for ADCs throughout the formulation development workflow, spanning from the initial formulation development to the final stage of drug product manufacturing. In addition, we identify and analyze the distinctive technical challenges in ADC formulation development compared to unconjugated antibody, while proposing potential solutions to these challenges. Finally, we offer strategic perspectives on future directions in ADC formulation development to advance this promising therapeutic modality.

## Teaser

Statement of Significance: This article comprehensively reviews and summarizes the formulations of commercial antibody–drug conjugates (ADCs) for the first time. The discussion extends to essential factors in drug product manufacturing and logistics processes. Additionally, the unique technical challenges of ADC formulation development compared to unconjugated antibody and the corresponding solutions are elucidated.

## Introduction

Antibody–drug conjugates (ADCs), also known as the “biological missiles,” are an innovative class of biological therapeutics. Structurally, ADC is composed of three key elements: a monoclonal antibody (mAb) for target specificity, a cytotoxic drug payload, and a specialized linker connecting these two agents [1]. The combination of these three components synergistically enhances the selectivity and efficacy of antitumor products, offering ADC superior therapeutic outcomes than traditional treatments. As of December 2024, 16 ADCs for oncology treatment have been approved by regulatory agencies in the world for marketing. Furthermore, the therapeutic potential of ADC continues to expands, with over 370 novel ADC candidates currently undergoing clinical evaluation across both oncological and non-oncological indications [2].

The development of robust formulations is crucial in transforming an experimentally effective ADC into commercially viable therapeutics. An optimal formulation should maintain the stability of active pharmaceutical ingredients, mitigate physicochemical degradation during product distribution and storage, and ultimately ensure optimal therapeutic performance at the target site [3]. As detailed in Table 1, which provides a comprehensive overview of formulation parameters for commercial ADC products, key development considerations include dosage form, dose range, concentration, excipient composition, primary packaging configuration, and molecular characteristics. In addition, the implementation of effective drug product manufacturing, coupled with optimized distribution and administration practices, is essential to ensure that ADC products are delivered safely and reliably to healthcare facilities and patients [4]. This review systematically examines critical aspects of ADC formulation development and highlights pivotal considerations for manufacturing and logistics processes.

**Table 1:** Formulation information of commercial ADC products [5–7]

**Product name**	**Approval**	**Target**	**Indication**	**Dose**	**Dosage form, strengths**	**Storage condition, shelf life**	**ROA**	**Formulation composition (post recon for lyo product)**	**CCS**	**DAR**	**Conjugation technique**	**Linker**	**Payload**
Gemtuzumab ozogamicin (**Mylotarg**®)	2000/2017	CD33	Leukemia	3 mg/m^2^	Lyo, 4.5 mg/vial	2°C–8°C 60 months	IV	1 mg/ml ADC, 5 mM sodium phosphate, 99.25 mM NaCl, 0.9% dextran 40, 1.55% sucrose, pH 7.5	Amber glass vial	2–3	Lysine coupling	Hydrazone	Calicheamicin
Brentuximab vedotin (**Adcetris**®)	2011	CD30	Lymphoma	1.8 mg/kg	Lyo, 50 mg/vial	2°C–8°C 48 months	IV	5 mg/ml ADC, 20 mM sodium citrate, 7% trehalose, 0.02% PS80, pH 6.6	Glass vial	~4	Cysteine coupling	MC-VC-PABC	MMAE
Ado-trastuzumab emtansine (**Kadcyla**®)	2013	HER2	Breast cancer	3.6 mg/kg	Lyo, 100 mg or 160 mg/vial	2°C–8°C 48 months	IV	20 mg/ml ADC, 10 mM sodium succinate, 6% sucrose, 0.02% PS20, pH 5.0	Glass vial	~3.5	Lysine coupling	MCC	DM1
Inotuzumab ozogamicin (**Besponsa**®)	2017	CD22	Leukemia	0.5, 0.8 mg/m^2^	Lyo, 0.9 mg/vial	2°C–8°C 60 months	IV	0.25 mg/ml ADC, 20 mM Tris–HCl, 10 mM NaCl, 5% sucrose, 0.01% PS80, pH 8.0	Amber glass vial	~6	Lysine coupling	Hydrazone	Calicheamicin
Moxetumomab pasudotox-tdfk (**Lumoxiti**®)	2018	CD22	Leukemia	0.04 mg/kg	Lyo, 1 mg/vial	2°C–8°C 48 months	IV	1 mg/ml ADC, 25 mM sodium phosphate, 8% glycine, 4% sucrose, 0.02% PS80, pH 7.4	Glass vial	NA	Genetically fused	NA	PE38
Polatuzumab vedotin-piiq (**Polivy**®)	2019	CD79b	Lymphoma	1.8 mg/kg	Lyo, 140 mg/vial	2°C–8°C 30 months	IV	20 mg/ml ADC, 10 mM Succinic acid, 14 mM sodium hydroxide, 4% sucrose, 0.12% PS20, pH 5.3	Glass vial	~3.5	Cysteine coupling	MC-VC-PABC	MMAE
Enfortumab vedotin-ejfv (**Padcev**®)	2019	Nectin-4	Urothelial cancer	1.25 mg/kg	Lyo, 20 mg or 30 mg/vial	2°C–8°C 36 months	IV	10 mg/ml ADC, 20 mM His·HCl, 5.5% trehalose, 0.02% PS20, pH 6.0	Glass vial	~4	Cysteine coupling	MC-VC-PABC	MMAE
Fam-trastuzumab deruxtecan-nxki (**Enhertu**®)	2019	HER2	Breast cancer	5.4 mg/kg	Lyo, 100 mg/vial	2°C–8°C 48 months	IV	20 mg/ml ADC, 25 mM His·HCl, 9% sucrose, 0.03% PS80, pH 5.5	Amber glass vial	~8	Cysteine coupling	MC-GGFG	DXd
Sacituzumab govitecan-hziy (**Trodelvy**®)	2020	Trop-2	Triple-negative breast cancer	10 mg/kg	Lyo, 180 mg/vial	2°C–8°C 36 months	IV	10 mg/ml ADC, 20 mM MES, 0.86% trehalose, 0.01% PS80, pH 6.5	Glass vial	~7.6	Cysteine coupling	CL2A (MCC-PEG-carbonate)	SN-38
Belantamab mafodotin-blmf (**Blenrep®**)	2020	BCMA	Myeloma	2.5 mg/kg	Lyo, 100 mg/vial	2°C–8°C 48 months	IV	50 mg/ml ADC, 25 mM sodium citrate, 0.2 M trehalose, 0.002% EDTA, 0.02% PS80, pH 6.2	Glass vial	~4	Cysteine coupling	MC	MMAF
Cetuximab sarotalocan sodium (**Akalux**®)	2020	EGFR	Head and neck cancer	640 mg/m^2^	Liquid, 250 mg/vial	2°C–8°C 18 months	IV	5 mg/ml ADC, 10 mM sodium phosphate, 9% trehalose, 0.02% PS80, pH 7.1 ± 0.5	Amber glass vial	2–3	Lysine coupling	Aminocaproic acid	Irdye 700DX
Loncastuximab tesirine-lpyl (**Zynlonta**®)	2021	CD19	Lymphoma	0.075, 0.15 mg/kg	Lyo, 10 mg/vial	2°C–8°C 48 months	IV	5 mg/ml ADC, 20 mM His·HCl, 6% sucrose, 0.02% PS20, pH 6.0	Glass vial	~2.3	Cysteine coupling	MC-PEG-VA-PABC	PBD dimer
Disitamab vedotin (**Aidixi**®)	2021	HER2	Gastric cancer	2.5 mg/kg	Lyo, 60 mg/vial	2°C–8°C 24 months	IV	10 mg/ml ADC, His·HCl/NaOH, sucrose, mannitol, PS80	Glass vial	4	Cysteinev coupling	MC-VC-PABC	MMAE
Tisotumab vedotin-tftv (**Tivdak**®)	2021	TF	Cervical cancer	2 mg/kg	Lyo, 40 mg/vial	2°C–8°C 36 months	IV	10 mg/ml ADC, 30 mM His·HCl, 3% sucrose, 3% mannitol, pH 6.0	Glass vial	4	Cysteine coupling	MC-VC-PABC	MMAE
Mirvetuximab soravtansine (**Elahere**®)	2022	FRα	FRα positive tumor	6 mg/kg	Liquid, 100 mg/vial	2°C–8°C 60 months	IV	5 mg/ml ADC, 10 mM sodium acetate, 9% sucrose, 0.01% PS20, pH 5.0	Glass vial	~3.4	Lysine coupling	Sulfo-SPDB	DM4
Sacituzumab tirumotecan (**sac-TMT**®)	2024	Trop-2	Triple-negative breast cancer	5 mg/kg	Lyo, 200 mg/vial	2°C–8°C 12 months	IV	20 mg/ml ADC, His/His·HCl, sucrose, PS20	Glass vial	~7.4	Cysteine coupling	Sulfonyl pyrimidine-CL2A-carbonate linker	KL610023 (Topoisomerase I inhibitor)

Abbreviations: ROA, Route of administration; Lyo, Lyophilization; CCS, Container Closure System; DAR, Drug-to-antibody Ratio; IV, Intravenous; His, Histidine; MES, 2-(*N*-morpholino) ethane sulfonic acid; Tris, Tris(hydroxymethyl)aminomethane; EDTA, Ethylenediaminetetraacetic acid; PS80, Polysorbate 80; PS20, Polysorbate 20; MCC, Maleimidomethyl cyclohexane-1-carboxylate; MC-VC-PABC, Maleimidocaproyl-Valine-citrulline-*p* aminobenzyl carbamate; MC-GGFG, maleimidocaproyl glycine–glycine-phenylalanine-glycine linker; MC, Maleimidocaproyl; MC-PEG-VA-PABC, Maleimidocaproyl-PEG-Valine-alanine-*p*-aminobenzyl carbamate; sulfo-SPDB, 1-(2,5-dioxopyrrolidin-1-yl)oxy-1-oxo-4-(pyridin-2-yldisulfanyl)butane-2-sulfonic acid; MMAE, Monomethyl auristatin E; MMAF, Monomethyl auristatin F; PE38, Pseudomonas exotoxin; DXd, Exatecan derivative; SN-38, 7-Ethyl-10-hydroxycamptothecin; PBD, Pyrrolobenzodiazepine; DM1, N2′-Deacetyl-N2′-(3-mercapto1-oxopropyl)-maytansine; DM4, N2′-Deacetyl-N2′-(4-mercapto-4-methyl-1-oxopentyl)-maytansine.

Note: 1. **Mylotarg**® was firstly approved by FDA in 2000, withdrawn in 2011, re-approved in 2017. **Blenrep**^**®**^ was withdrawn from US and EU markets. 2. For lyo products, concentrations refer to the concentrations after reconstitutions. 3. **Lumoxiti**^**®**^, as an immunotoxin, is indeed a recombinant antibody genetically fused with a toxic protein, and was already withdrawn from the market.

## Principal focus points in ADC formulation development

**Figure 1:**
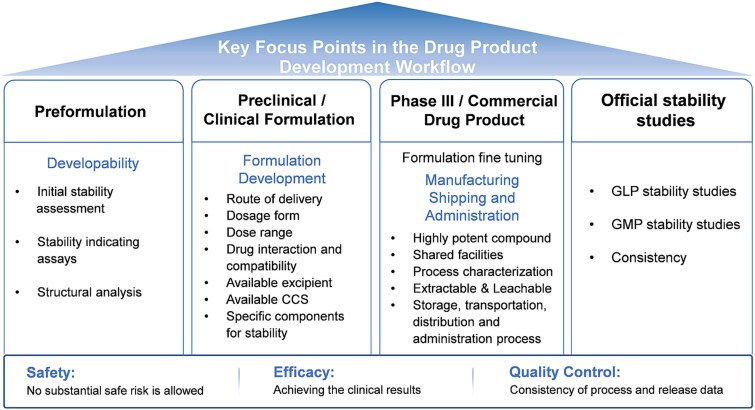
Principal focus points in the ADC drug product development framework.

The primary objective of drug product development is to design and manufacture safe, effective, and quality products to be consistently delivered and meet the medical needs. A high-level formulation development and manufacturing roadmap from early stage to commercialization are summarized in Fig. 1. The subsequent sections will provide an in-depth analysis of the key technical considerations and strategic decision points identified in this developmental pathway.

### Developability

Developability is the likelihood that an ADC candidate will go smoothly through the chemistry, manufacturing, and controls (CMC) process to become a safe, efficacious, and manufacturable drug [8]. The assessment of ADC developability requires comprehensive evaluation of both antibody-related characteristics (including structural homogeneity, thermal stability, solubility, and target specificity) and linker-payload physicochemical properties. These parameters must be considered in conjunction with the intended clinical application and administration route. For example, “hotspots” on the antibody that may be deleterious for stability as well as for pharmacokinetic and pharmacological properties was identified and antibodies with low mannose content was selected to reduce the potential off-target hepatic toxicities [9]. As analysed in a published article [10], only two payloads used in commercially approved ADCs, DXd, and SN-38 are qualified for the physicochemical characteristics as defined by the well-known Lipinski rules for drug-likeness, while some of other payloads have limitations when penetrating biological membranes.

Furthermore, physicochemical properties of ADC, including viscosity, solubility, melting temperature, isoelectric point, and hydrophobicity profile, can be optimized through linker-payload chemistry with significantly improved solution stability for subcutaneously dosing [11]. The development of subcutaneous formulations for ADCs such as sacituzumab govitecan (IMMU-132) and related compounds (IMMU-130 and IMMU-140) is constrained by concentration-dependent challenges, including protein aggregation and precipitation, which limit maximum achievable dosing volumes [12]. To address these challenges, early-stage developability assessment employs high-throughput screening methodologies, enabling efficient evaluation of multiple candidates with limited material availability [13]. This developability study can be used to gain preliminary understanding of the characteristics and properties of multiple ADC candidates to support the lead molecule selection for further development.

### Route of delivery

During the development of ADC formulation, the delivery route needs careful consideration of patient compliance, clinical efficacy, and safety profiles. Antibody administration has traditionally relied on parenteral routes, including intravenous (IV) infusion, subcutaneous (SC) injection, intravenous bolus, intravitreal, and intramuscular delivery, while alternative approaches such as oral and nasal delivery system are currently under active investigation [14–16]. As shown in Table 1, all approved ADCs are intended for oncology indications and administrated only via IV infusion in a healthcare provider setting. At present, other delivery routes are rarely applied in late clinical stage due to the local deposition and toxicity of the payload [17–19]. Current clinical trial data reveal that 55% of ongoing ADC studies target solid tumors, 44% focus on hematological malignancies, and 1% explore non-oncology indications [20]. However, with the development of ADCs for chronic diseases and SC delivery for antitumor drugs, SC administration is the most attractive alternative to IV injection as it provides several advantages such as self-administration, reduced treatment burden, improved patient compliance, reduced infusion-related reactions, and treatment for patients with poor venous access [21].

Despite setbacks in SC formulations such as Silverback’s SBT6050 (oncology) and AbbVie’s ABBV-154 (autoimmune diseases), which were discontinued due to efficacy and safety concerns, promising developments continue to emerge. Heidelberg Pharma’s ATAC®-based ADC (HDP-103) has demonstrated encouraging preclinical results, while Alphamab’s JSKN033, a novel co-formulation combining a HER2-targeting biparatopic ADC with a PD-L1 inhibitor, was advanced to phase I/II clinical trials [22–24]. The preliminary clinical result of JSKN033 was reported as an abstract at an international conference in the United States in November 2024 [25]. Recently, a subcutaneous version of trastuzumab deruxtecan, which employs payload with modest potency/toxicity and optimized hydrophobicity, is being developed through co-formulation with the human hyaluronidase, ALT-B4. As summarized in Table 2, the development of SC formulations demonstrates a preferential selection of payloads characterized by reduced hydrophobicity. Overall, it seems to us that there is an increasing interest from biopharmaceutical industry in exploring subcutaneous ADCs for both oncology and non-oncology indications.

**Table 2:** Hydrophobicity data of payload and linker-payload for ADCs administered intravenously and subcutaneously

**Route of administration**	**ADC**	**CLogP** ^**a**^ **of payload**	**CLogP** ^**a**^ **of linker-payload**
SC development	Enhertu®	0.2	−0.3
	JSKN003	0.2	1.6
	ABBV-154	−0.6	1.2
	HDP-103	−3.2	−1.0
IV approved	Mylotarg®, Besponsa®	1.8	3.6
	Adcetris®, Polivy®, Padcev®	4.9	5.7
	Blenrep®	2.9	6.0
	Kadcyla®	4.2	4.6
	Elahere®	4.9	3.9
	Zynlonta®	2.7	2.1
	Enhertu®	0.2	−0.3
	Trodelvy®	1.7	−0.1
	Sac-TMT®	0.8	−2.1

^a^Partition coefficients (CLogP value) were calculated using ChemDraw 20.0 software.

### Dosage form and dose range

Considering parenteral administration as the main delivery route of biological drugs, liquid formulations are preferred and about two-third of the injected products on the market are solutions because of its lower costs and more convenience for use than lyophilized drug products [3, 26]. However, the ADC formulations in liquid or frozen liquid form may encounter more stability issues and shorter shelf-life than lyophilized versions. An optimal formulation development for an ADC should not only take into account the mAb stability, but also consider the chemical stability of the linker and payload [27]. As shown in Table 1, 14 out of 16 commercial ADCs are lyophilized and 2 (i.e. Elahere® and Akalux®) of 16 commercial ADCs are formulated in solutions with buffers, sugars, and surfactants. Advancements in innovative linker-payload technology and a better understanding of linkers, payloads, and antibody components can enable ADC developers to adopt informed formulation strategies for determining the dosage form of ADCs. This progress can facilitate the transition from lyophilized products to liquid formulations without significantly delaying the product development timeline [4].

In addition, dose for ADC can be quite low (e.g. Inotuzumab ozogamicin at 0.5 mg/m^2^, Moxetumomab pasudotox-tdfk at 0.04 mg/kg, and Loncastuximab tesirine-lpyl at 0.075 mg/kg). These extreme low dosages require significant levels of dilution of the ADC drug products, resulting in the risks of adsorptive losses, increased aggregation and difficulties in analyzing and assessing the quality parameters at the low concentrations [28]. Surface adsorption caused by payload hydrophobicity and particle formation during dilution can be ameliorated to some extent by the addition of IV solution stabilizer [29]. All the above-mentioned factors associated with dosage form and dose range need to be fully considered during the formulation development for ADC products.

### Drug interaction and compatibility study

Since ADCs have unique structures with both small and large molecule components, each ADC typically has its own distinct pharmacokinetic property *in vivo*. The theoretical drug interaction (DI) mechanism for an ADC is related to both the antibody molecule and its cytotoxic payload [30]. Notably, two widely utilized payload classes—monomethyl auristatin E (MMAE) and maytansine (DM1)—undergo primary metabolism via cytochrome P450 3A4 (CYP3A4). Co-administration of MMAE-based ADC (including Adcetris®, Polivy®, Padcev®, Aidixi®, and Tivdak®) or DM1-based ADC (such as Kadcyla®) with potent CYP3A4 inhibitor may lead to increased exposure of payload and toxicity [31]. It is also reported that co-administration of strong CYP3A inhibitor with multiple doses of Enhertu® can elevate the steady-state exposure (AUC_0–17 days_), but the impact of these changes is not clinically meaningful.

ADC and immune-oncology (ADC/IO) combination therapy has become a major focus of the current preclinical and clinical studies in the past several years. Majority of commercially approved ADCs except for two ADCs (i.e. inotuzumab ozogamicin and the discontinued moxetumomab pasudotox) are under clinical trials in combination with immune checkpoint modulators [32]. This therapeutic strategy leverages the complementary mechanisms of action: ADC increases the infiltration of T cells into the tumor microenvironment and then immune-checkpoint inhibitors reinvigorate exhausted T cells. This potential synergy mechanism may be achieved through two combination approaches, i.e. co-administration and co-formulation. The compatibility study for co-administrated ADC is focused on clinical in-use process. Generally, co-administration via the same bag or infusion system is not recommended in the clinical use due to lack of knowledge of chemical or physical compatibility with other drugs [33]. In contrast, co-formulation development requires comprehensive evaluation of long-term stability and potential DIs throughout the product shelf life [34]. A representative example is the development of hyaluronidase-based co-formulations, which necessitates careful assessment of mutual compatibility. The enzymatic component should not interfere with the drug release mechanism, critical quality attributes, degradation profile, and safety characteristics of ADC compound. Conversely, the ADC formulation should maintain hyaluronidase activity, with both components demonstrating stable coexistence in the combined product [35].

### Excipients and container closure systems for ADCs

Table 1 summarizes excipients commonly employed in commercial ADC formulation, including buffer system, lyoprotectants, and surfactants. Marketed ADCs predominantly utilize pH ranges of 5.0–8.0, with buffer systems such as histidine, MES, citrate, phosphate, Tris, succinate, and acetate selected to match these conditions. For instance, Mylotarg® and Besponsa® employ phosphate (pH 7.5) and Tris (pH 8.0) buffers, respectively, to protect the acid-sensitive hydrazone bonds in their linkers. Similarly, Trodelvy® incorporates carbonate-based CL2A linker, which exhibits pH-dependent drug release in tumor microenvironments, its formulation adopts relatively high pH (pH 6.5) to optimize stability [36, 37]. While acetate buffers demonstrate effective buffering capacity in the low pH range for liquid formulations, their volatility and potential sublimation limit their application in lyophilized products, as evidenced by their selective use in Elahere® [38]. Lyoprotectants, in particular disaccharides, are preferentially excluded from the ADC surface to stabilize the protein both in aqueous solutions and lyophilization process [39]. Surfactants (e.g. polysorbate 20 or 80 at 0.1–2 mg/ml) were added in ADC formulation to migrate the denaturation at air–water interfaces or under mechanical stresses. Antioxidant strategies include methionine supplementation to scavenge free thiols, alongside chelating agents such as ethylenediaminetetraacetic acid and diethylenetriaminepentaacetic acid to inhibit metal-catalyzed destabilization [40].

Lyophilization remains the preferred formulation strategy for most ADCs, both in commercial products and at clinical-stage candidates, due to its ability to preserve conjugate integrity and minimize payload premature release during storage. However, the lyophilization process generates the freezing and drying stresses, such as solute concentration, formation of ice crystals, and pH changes, which can denature proteins to various degrees. Consequently, appropriate excipients for lyophilized formulation should be carefully selected at the early stage of ADC formulation development. In general, ionic excipients lower the glass transition temperature (Tg') of the formulation and are not recommended or kept to a minimum, while disaccharides with intermediate and tolerated Tg' value are frequently used as lyoprotectants. Biopolymers, including proteins themselves, raise the Tg' and have been reported to stabilize proteins and make more efficient lyophilization cycles possible [41].

Per regulatory definitions, a novel excipient refers to any inactive ingredient first introduced into pharmaceutical products or applied via a new route of drug delivery [42]. For example, MES buffer enhances photostability in polymer dots (Pdots) through radical scavenging mechanisms [43]. To date, MES buffer was only used in Trodelvy® (SN38-based ADC) as a novel excipient and releasing tested with an in-house quality standard. Sulfobutylether β-cyclodextrin sodium salt is a class of cyclodextrins which acts as a stabilizer and solubilizer of ADC active substances. Cyclodextrins can prevent agitation-associated aggregation in solution as well as lyophilization-related aggregation through reducing attractive protein–protein interactions and liquid–liquid phase separation [44, 45]. As subcutaneous ADC formulations advance clinically, future development may necessitate viscosity-lowing excipients (e.g. ionic salts, amino acids such as arginine, glycine, proline, and lysine, or caffeine) alongside recombinant human hyaluronidase to optimize drug delivery [17, 46, 47].

Innovative container closure systems have been developed to address specific formulation challenges in ADC drug products. Advanced surface modification technologies, including plasma impulse chemical vapor deposition and baked-on coating methods, have demonstrated significant improvements in reducing lyophilization fogging and solution residual emptying [48, 49]. For photosensitive drugs, amber glass vial and light protection label can be applied to protect the drug from light throughout storage and handling.

### Specific components for ADC stability

**Figure 2:**
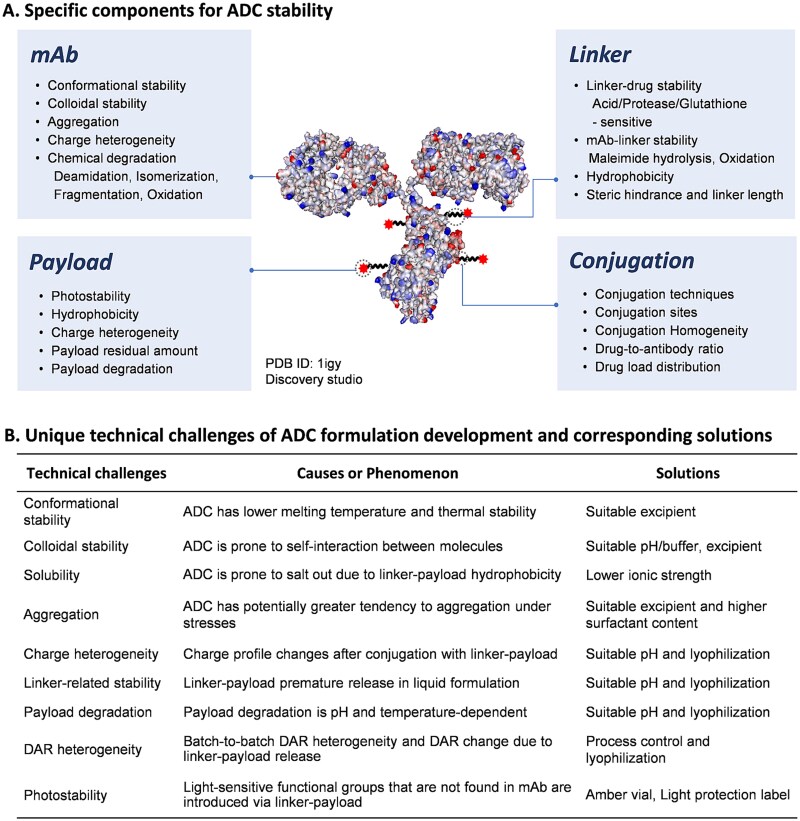
Specific components for ADC stability and unique technical challenges of ADC formulation development. (A) ADC stability assessment encompasses multiple critical dimensions: the integrity of the monoclonal antibody (mAb), linker, and payload as individual components, as well as the stability of the fully conjugated assembly. (B) The corresponding solutions to the unique technical challenges of ADC formulation development compared with mAb have been proposed.

Due to the structural complexity of ADC, the potential specific stability issues during the formulation development should be considered to ensure the efficacy of ADC products. As illustrated in Fig. 2A, ADC stability profiling can be approached from a number of different perspectives, including the stability of the individual linker, payload and mAb components as well as the stability of the entire conjugation assembly. Each specific component for ADC stability is discussed in the following subsections.

#### mAB component

In an ADC molecule, the mAb component represents the biologic targeting agent and serves as the proteinaceous body. Although ADC and mAb share comparable secondary and tertiary structures, ADC typically exhibits diminished conformational stability and colloidal stability relative to mAb [50]. Lower melting temperature is observed for ADC as they are more prone to be destabilized by thermal stress. What's more, more hydrophobic surface around the conjugation site in the ADC molecule has been detected indicating its greater tendency to aggregate [51]. In terms of protein, especially for ADC, its salting-in (increased solubility) or salting-out (decreased solubility) behaviors are impacted by the ionic strength in formulation development. Elevated ionic concentrations attenuate charge repulsion forces, potentially fostering protein–protein attractions that reduce colloidal stability—a relationship quantifiable through parameters like k_D_ and B_22_ [52]. Similar to the naked mAb, conjugated mAb component is susceptible to chemical degradation pathways like deamidation, isomerization, fragmentation, and oxidation, depending on the pH/buffer environment [53]. These types of degradations lead to change in hydrophobicity, charge heterogeneity or induced aggregation. If found in the complementary-determining region, these degradations may also impact product potency.

#### Linker component

As the linker in an ADC is the bridge connecting the mAb and the payload, it should have sufficient systemic circulatory stability while maintaining appropriate conditional instability for triggered payload release [54]. So, ADC stability can be markedly influenced by the chemical properties of designed linker or the linker-related instability, which can be subdividable into *linker-drug instability* and *mAb-linker instability* [2]. Table 3 summarizes conjugation technologies and linker stability studies of approved ADC products. As shown in Table 3, the approved ADCs are considered to have certain levels of linker-related instabilities whether it is non-cleavable or cleavable based on the ADCs' *in vitro* stability data. One example is that the level of released free drug reached a plateau of 5%–6% of total drug following 2 months of storage at 15°C in liquid solution [55].

**Table 3:** Summary of approved ADC linker technologies and linker stability studies

**Linker-payload**	**Type**	**ADC**	**Estimation of antibody-linker instability in circulation** ^**a**^ [2]	**Estimation of linker-payload instability in circulation** ^**b**^ [2]	**ADC *in vitro* stability (plasma)**	**ADC *in vitro* stability (buffer)**
Ozogamicin	Cleavable	Mylotarg® Besponsa®	/	Plasma 7 days: 20% remaining	Low plasma stability [54]	pH 4.5, 37°C 24 hrs: 97% hydrolysis [65] pH 7.4, 37°C 24 hrs: 6% hydrolysis
Vedotin	Cleavable	Adcetris® Polivy® Padcev® Aidixi® Tivdak®	Plasma 7 days: 50% remaining	/	Plasma 10 days: 2% free drug release [66]	Adcetris® (CS: DAR 3.5, Free drug 0.3 μg/ml^c^) [67] pH 4.0, 37°C 4 weeks: DAR 1.3, Free drug 110 μg/ml^c^ pH 6.0, 37°C 4 weeks: DAR 3.2 pH 8.0, 37°C 4 weeks: DAR 2.2 pH 10.0, 37°C 4 weeks: DAR 2.1, Free drug 70 μg/ml^c^
Emtansine	Non-Cleavable	Kadcyla®	/	Plasma 7 days: 50% remaining	Plasma 4 days: 70% remaining [68]	Kadcyla® (CS: 0.47% free maytansinoid) [69] pH 4.0, 37°C 4 weeks: 2.4% free maytansinoid pH 10.0, 37°C 4 weeks: 6.1% free maytansinoid
Deruxtecan	Cleavable	Enhertu®	Plasma 7 days: 50% remaining	/	Plasma 21 days: 2.1% free drug release [70]	PB with 1% BSA, 37°C 21 days: 0.2% free drug release, comparable or rather lower than Kadcyla® and Adcetris® [70]
Govitecan	Cleavable	Trodelvy®	Plasma 7 days: 50% remaining	Plasma 7 days: 50% remaining	Plasma 36 hrs: 50% free drug release [71]	pH 5.3, 37°C 13 hrs: 50% SN-38 release pH 7.4, 37°C 30 hrs: 50% SN-38 release [37]
Tirumotecan	Cleavable	sac-TMT®	/	Plasma 7 days: 50% remaining	Plasma 144 hrs: 70% free drug release [72]	No formulation stability data is disclosed.
Tesirine	Cleavable	Zynlonta®	Plasma 7 days: 80% remaining	/	Stable in plasma [73]	pH 7.8, 37°C 3 days: decrease (~0.11 units) of isoelectric point [74]
Soravtansine	Cleavable	Elahere®	/	Plasma 7 days: 40% remaining	Stable in plasma [75]	Soluble in neutral buffer and stable for up to 2 years upon storage at 4°C [76]

^a^ Antibody-linker instability in circulation refers to the whole linker-drug release from the antibody, such as the retro-Michael reaction for thiol–maleimide conjugation.

^b^ Linker-payload instability in circulation refers to the drug release over time prior to its intended site of metabolism.

^c^ Free drug includes MMAE, NACvcMMAE and other unknown free drugs.

Abbreviation: CS, control sample.

Note: Blenrep® and Lumoxiti® were withdrawn from markets. Akalux® employs a unique linker system distinct from conventional ADCs. These three ADCs are excluded from Table 3.

Succinimide ring hydrolysis and maleimide exchange, observed both *in vitro* and *in vivo*, remain critical quality concerns for ADCs [56]. Up to now, 10 out of the 16 commercially available ADCs employ cysteine–maleimide Michael addition for antibody–payload conjugation. The succinimide ring in the thiol–maleimide linker is susceptible to ring-opening reactions via hydrolysis, especially at high pH and elevated temperatures [57]. While this ring-opened hydrolyzed succinimide does not trigger retro-Michael exchange or premature payload release, it introduces additional charge heterogeneity. To mitigate this, structural modifications, including PEG groups, basic amines, aryl rings, dioxane moieties, and variable-length carbon chains, are strategically positioned near the maleimide group to accelerate hydrolysis rates [58].

Oxidation in linkers is another concern as it could affect the linker stability. The thioether succinimide linker was reported to be oxidized in a mild aqueous environment followed by sulfoxide elimination, particularly in the presence of hydrogen peroxide (H_2_O_2_), high pH and temperature. For mAb-SMCC-DM1 conjugates, free maytansinoid (DM1-${\text S}{\text O}_{\text 3}^{-}$) release exhibited H_2_O_2_ concentration-dependent kinetics (9.8 μM ˃ 3.3 μM ˃ 1.1 μM), accelerated rates at pH 7.4 vs. pH 5.5, and with a longer half-life for thio-succinimide cleavage from sulfoxide at 20°C vs. 37°C [59].

In the harsh systemic circulation environment, steric hindrance provides a good physical protection. For example, disulfide linker is thermodynamically stable at physiological pH in the absence of free sulfhydryl groups. It was further stabilized through steric hindrance by introducing two methyl groups adjacent to the disulfide to amplify steric shielding [60]. Moreover, a shorter linker typically comes to better ADC stability by shielding the payload further inside the local conformation of the antibody relative to a longer linker [11, 61].

To address the hydrophobic nature of payloads, hydrophilic linkers have been engineered to carry a larger number of hydrophobic payload per antibody without triggering aggregation or loss of affinity [62]. The design strategy integrates several key elements: hydrophilic moieties (β-glucuronide, polyethylene glycol spacers), charged groups (sulfonate, phosphate/pyrophosphate), terminal polar residues (taurine, amino, and sugar groups), and optimized dipeptide spacer (Ala-Ala/Gly-Glu as alternatives to Val-Cit/Val-Ala), which enables the higher solubility of ADC [24, 62–64].

#### Payload component

One of the fundamental differences between ADC and naked mAb is the presence of potent drug payload. Thus, several stability-related properties specific to payload, such as photostability, hydrophobicity, and payload degradation, should be considered in formulation development process.

Light exposure poses a common concern with ADC molecules as the payload component can undergo photodegradation, impacting the overall stability of the drug. Approved mAb products are normally packaged in clear vials while three commercial ADC drugs (Mylotarg®, Besponsa®, and Enhertu®) approved by FDA and one commercial ADC drug (Akalux®) approved by Pharmaceuticals and Medical Devices Agency (PMDA) in Japan are packaged in amber vials, as documented in Drugs@FDA database [77]. Specifically, light-sensitive functional groups that are not found in naked mAb are introduced via linker-payload conjugation in some types of ADCs such as calicheamicins, exatecan, anthracyclines, duocarmycins, porphyrins, chlorines, and bacteriochlorins [78]. Calicheamicin derivatives and their conjugates undergo rapid degradation under UV irradiation, as confirmed through experimental studies using both low-intensity UV lamps and natural sunlight [79, 80]. Similarly, camptothecin and its derivatives exhibit significant photodegradation characteristics, manifested by the formation of cloudy yellow solutions with visible precipitation when exposed to laboratory lighting or sunlight, particularly in neutral and alkaline conditions [81, 82].

In addition to the photodegradation, pH-dependent (5, 7, 9) and temperature-sensitive (8°C, 25°C, 40°C) degradation is commonly observed on ADC payloads as well, with higher temperature and lower pH lead to worse degradation [83]. Unwanted and unexpected toxicity resulting from ADC degradations may occur, potentially compromising therapeutic efficacy and patient safety.

As mentioned previously, hydrophobicity is one of the most important physicochemical properties of small molecule drugs, which is highly related with enhanced *in vitro* potency, poor solubility, metabolic instability, and nonspecific off-target effects. Payload-dependent structure destabilization and enhanced propensity to self-aggregation driven by increasing payload hydrophobicity was observed in the ADC process development stage [84]. For small molecule drugs, increased hydrophobicity comes with the risk of poor aqueous solubility. For ADC molecules, linker-payload hydrophobicity was experimentally proven to be an important factor leading to aggregation, but not the only driver for ADC instability and shelf life. What's more, high ionic strength formulations often show poorer properties and accelerate aggregation of partially unfolded species compared to low ionic strength conditions [13].

#### Conjugation characteristics

Beyond the structural components previously outlined, conjugation characteristics and high levels of homogeneity in bioconjugation are crucial to CMC quality control. Notably, several biophysical properties can be influenced by the conjugation characteristics, including conjugation techniques employed, conjugation sites, drug-to-antibody ratio (DAR) value, and drug load distribution. Rigorous characterization of these factors is essential to ensure batch consistency and therapeutic performance.

Nowadays, conjugation techniques varied from lysine–amide coupling, cysteine–maleimide alkylation, non-canonical amino acid engineering, disulfide re-bridging, and enzymatic coupling to glycoconnecting. Studies demonstrate that lysine-based conjugates showed greater agitation-induced aggregation compared to interchain cysteine conjugates [85]. Non-natural amino acid-enabled site-specific conjugation technology exhibits high homogeneity, stability, and extended half-life [86]. Various enzyme-mediated approaches instead of traditional maleimide coupling, such as sortase-mediated conjugation, transglutaminase-mediated conjugation, formylglycine-generating enzyme (SMARTag™), enable ADC molecules with high stability in serum across species under physiological conditions (37°C) [87]. Speaking of the site-specificity, conjugation site represents the local structural environment, such as solvent accessibility and charged environment, should be carefully selected since it is related to essential ADC properties including safety, activity, stability, and pharmacokinetics [88, 89]. For example, the hydrolysis of the succinimide ring in the linker was promoted when conjugated to cysteine on the light chain (LC) compared to the heavy chain (HC), attributed to the LC's solvent-exposed, positively charged microenvironment [90].

DAR and drug load distribution are vital to ADC product quality. To investigate the stability impact of individual DAR species, ADC fractions isolated via semipreparative hydrophobic interaction chromatography were incubated at high temperature in the presence of varying saline conditions (low vs. high ionic strength). The results showed that ADCs with higher DAR may be more susceptible to the formation of soluble and insoluble aggregates in saline environments due to salting-out effects and enhanced hydrophobic interaction [91]. Optimizing conjugation processes to increase the proportion of DAR4 species (four payload molecules per mAb) is an important strategy for improving homogeneity in cysteine-linked ADCs [92]. One example is WuXi XDC's proprietary WuXiDAR4™ technology. This platform and enrichment technology greatly enhances DAR4 percentage in the final ADC product and improves conjugation efficiency. Precisely controlling DAR has been a major challenge for the ADC industry. WuXiDAR4™ technology tightly controls ADC product homogeneity allowing for more precise quality control of ADC molecules [92, 93].

## Manufacturing, shipping, and administration

Once the formulation development is complete in the non-GMP labs, numerous other obstacles must be addressed to produce a commercial-ready ADC product.

### DP manufacturing process

During manufacturing process development, the primary objective is to establish reproducible process to consistently deliver high quality products that meets acceptance criteria for all quality attributes [94]. DAR value is a critical parameter during the process development of ADCs, which directly affects the efficacy, safety, and stability of ADCs. For some ADCs, the final product is a heterogeneous mixture of antibodies containing a range in the number of drugs conjugated per molecule [94]. And the small molecule drugs used in ADC products are usually highly cytotoxic compounds, necessitating elevated standards and stringent controls within the manufacturing process [95]. The use of closed systems and automated equipment (specifically designed for high-potent drugs) and the implementation of strict safety control measures (the use of hydrogen peroxide-based technology to decontaminate/sterilize isolators or glove boxes) are the key points in the DP manufacturing process.

The adoption of single-use manufacturing technology in ADC production has been primarily driven by product safety considerations (mitigating cross contamination risks) and occupational exposure limits requirements. Plastic component systems used during DP manufacturing come into direct contact with ADCs and thus resulting in the generation and accumulation of process-related leachables. USP <1665> recommends standardized extraction protocol aimed at obtaining extractable profiles. The specified extraction solvents include acid extraction (pH 3), basic extraction (pH 10), and organic extraction (50% ethanol), with 40°C being selected as the working temperature [96].

### Storage, transportation, distribution, and administration process

Given the structural particularities and sensitivity of ADCs to efficacy, special attention must be paid to the following critical points during transportation and storage.

The potential for free drug release and degradation product formation during storage necessitates stringent temperature control. Transportation conditions, including extreme temperature fluctuations (e.g. increase to 15°C), may compromise structural integrity, leading to DAR value deviations. Additionally, repeated freeze–thaw cycles or lyophilization processes may result in measurable increases in free drug concentrations, while suboptimal freezing conditions may promote protein aggregation through multiple mechanisms: cryoconcentration effects, ice-liquid interfacial stresses, pH fluctuations, and phase separation phenomena [97]. Shaking could potentially induce protein aggregation although the extent of impact depends both on the intensity and duration of exposure to such stresses. Therefore, a suitable DS container closure system can mitigate the impact on ADC quality during transportation. The selection of containers for ADCs is subject to strict regulatory requirements designed to ensure the quality, stability, and safety of drugs. Common ADC DS containers options include: (i) polycarbonate (PC) and high-density polyethylene (HDPE) bottles with operational ranges extends to −90°C; (ii) polyethylene terephthalate glycol with brittleness temperature of −40°C; (iii) ethylene vinyl acetate bag: standard for cryogenic storage [98]. PC bottle talc particulates can be minimized by rinsing with water for injection in high-grade cleanrooms.

Owing to the cytotoxic properties of the payload, ADCs are generally classified as potentially hazardous drugs (HD) under the definition of the National Institute for Occupational Safety and Health [99]. Closed system transfer devices (CSTDs) provide critical protection during the compounding and administration process by creating a physical barrier that prevents environmental contaminants and HD exposure. The implementation of CSTDs necessitates comprehensive clinical in-use testing by pharmaceutical sponsors to verify both the physicochemical compatibility and dose recovery of the therapeutic agent. Critical evaluations must also address several potential risk factors: particulate generation from mechanical stress and device lubricants, drug loss through adsorption and residual holdup volume, and potential quality concerns including protein aggregation. Importantly, the incorporation of a 0.2 μm in-line filter is essential for ADC administration to maintain particulate levels within the USP <787> regulatory specifications.

## Conclusion

As an innovative form of drug modalities, ADCs possess tremendous clinical value and market potential in the realm of oncology therapeutics. This review highlights the fundamental properties and principal areas of focus in ADC formulation development as well as main points during the manufacturing and shipping. The administration route is a major consideration when designing the ADC formulation, high concentration ADCs and their co-formulations in SC delivery are currently explored although all commercial ADC drugs on the market are administrated intravenously.

Stable ADC formulations that are either lyophilized or in liquid form can be obtained through optimizing the developability of mAb and physicochemical characteristics of linker-payload, using innovative conjugation technologies and appropriate excipients and packaging materials. Furthermore, the risk of shared facilities, process robustness and consistency, extractable and leachable control, storage, and transportation should be paid special attention during ADC drug product manufacturing. The increased diversity in antibody conjugates calls for innovative excipient system, synergistic stabilizer combinations, and high-throughput stability indicating methods in ADC formulation development.

## Data Availability

The data that support the findings of this study are openly available.

## Animal research statement

This is not applicable.
